# D-Mannitol Induces a Brown Fat-like Phenotype via a β3-Adrenergic Receptor-Dependent Mechanism

**DOI:** 10.3390/cells10040768

**Published:** 2021-03-31

**Authors:** Hui-Jeon Jeon, Dong Kyu Choi, JaeHeon Choi, Seul Lee, Heejin Lee, Ji Hoon Yu, Sang-Hyun Min

**Affiliations:** New Drug Development Center, Daegu-Gyeongbuk Medical Innovation Foundation (DGMIF), 80 Chumbok-ro, Dong gu, Daegu 41061, Korea; hjjeon@dgmif.re.kr (H.-J.J.); dongkyu@dgmif.re.kr (D.K.C.); cjh0403@dgmif.re.kr (J.C.); autrition15@dgmif.re.kr (S.L.); free7e77@knu.ac.kr (H.L.); yujihoon@dgmif.re.kr (J.H.Y.)

**Keywords:** obesity, browning, D-mannitol, brown adipocyte, β3-adrenergic receptor

## Abstract

The presence of brown adipocytes within white adipose tissue is associated with phenotypes that exhibit improved metabolism and proper body weight maintenance. Therefore, a variety of dietary agents that facilitate the browning of white adipocytes have been investigated. In this study, we screened a natural product library comprising 133 compounds with the potential to promote the browning of white adipocytes, and found that D-mannitol induces the browning of 3T3-L1 adipocytes by enhancing the expression of brown fat-specific genes and proteins, and upregulating lipid metabolism markers. D-mannitol also increased the phosphorylation of AMP-activated protein kinase (AMPK) and acetyl-CoA carboxylase 1 (ACC), suggesting a possible role in lipolysis and fat oxidation. Moreover, an increase in the expression of genes associated with D-mannitol-induced browning was strongly correlated with the activation of the β3-adrenergic receptor as well as AMPK, protein kinase A (PKA), and PPARγ coactivator 1α (PGC1α). D-mannitol effectively reduced the body weight of mice fed a high-fat diet, and increased the expression of β1-oxidation and energy expenditure markers, such as Cidea, carnitine palmityl transferase 1 (CPT1), uncoupling protein 1 (UCP1), PGC1α, and acyl-coenzyme A oxidase (ACOX1) in the inguinal white adipose tissue. Our findings suggest that D-mannitol plays a dual regulatory role by inducing the generation of a brown fat-like phenotype and enhancing lipid metabolism. These results indicate that D-mannitol can function as an anti-obesity supplement.

## 1. Introduction

Obesity, a phenomenon that results from the accumulation of excess body fat, is caused by an imbalance in energy intake and output. As obesity leads to the development of several diseases, such as type 2 diabetes and metabolic syndrome [1], the dissipation of excessive calories into heat (non-shivering thermogenesis) via exercise is recommended to counter this condition [2]. A mouse model—in which the expression of the uncoupling protein 1 (UCP1) was induced—demonstrated that exercise increased not only mitochondrial biogenesis and activity, but also the presence of brown-like adipocytes.

Brown adipose tissue (BAT) and white adipose tissue (WAT) are associated with opposing functions. Compared with WAT, which stores excess energy as triglycerides, BAT contains multiple lipid droplets and more mitochondria. Further, BAT expresses UCP1 and is specialized for energy consumption via heat generation, thereby regulating thermo-homeostasis [3]. Therefore, the generation of a brown fat-like phenotype in WAT by inducing beiging or browning is considered an effective therapeutic strategy that helps to maintain energy homeostasis. Moreover, a recent discovery revealed that metabolically active BAT deposits in adults enable the utilization of glucose and fatty acids [4,5]. However, the activity of brown fat differs among individuals and disappears with aging [6]. These findings have resulted in the development of a new therapeutic perspective involving the treatment of obesity by activating BAT or increasing its mass [7].

UCP1 expression in response to external stimuli activates thermogenic potential by uncoupling the electron transport chain in the mitochondria of white fat depots [8]. The development of thermogenic beige adipocytes is markedly enhanced upon exposure to the cold or a β3-adrenergic receptor agonist [9]. Therefore, several studies have investigated the role of dietary supplements, such as capsaicin, berberine, and orexin, in β3-adrenergic receptor signaling, which induces the browning of WAT to increase energy expenditure and decrease fat accumulation in mammals [9,10,11].

D-mannitol is a naturally occurring six-carbon sugar alcohol or polyol that is widely distributed in plants, including algae, onions, grasses, and olives [12]. As D-mannitol has a relatively lower glycemic index than sucrose and is known to be safe, the US Food and Drug Administration (FDA) has approved the use of D-mannitol in patients with diabetes and in individuals with sugar intolerance. D-mannitol increases the production of short-chain fatty acids (SCFAs), which modulate lipid metabolism in the liver [13]. These findings suggest that D-mannitol also affects lipid and energy metabolism.

In this study, we searched for compounds that exhibit fat-browning activity by increasing the mitochondrial content, and identified a compound—D-mannitol—that induces the browning of differentiated 3T3-L1 adipocytes in high-fat diet-fed mice. Finally, we clarified the potential role of D-mannitol in fat browning, obesity, and lipid metabolism.

## 2. Materials and Methods

### 2.1. Cell Culture and Differentiation

Dulbecco’s modified Eagle’s medium (Thermo Fisher Scientific, Waltham, MA, USA) supplemented with 10% fetal bovine serum (Thermo Fisher Scientific) and 100 µg/mL penicillin–streptomycin (Thermo Fisher Scientific), was used to culture 3T3-L1 preadipocytes (ATCC, Manassas, VA, USA) at 37 °C in an incubator with an atmosphere of 5% CO_2_. Sufficiently confluent cells were maintained in a differentiation induction medium (10 µg/mL of insulin (Sigma Aldrich, St. Louis, MO, USA), 0.25 µM dexamethasone (Sigma Aldrich), and 0.5 mM 3-isobutyl-1-methylxanthine (IBMX, Sigma Aldrich)) in dulbecco’s modified eagle medium (DMEM), followed by DMEM supplemented with 10% fetal bovine serum (FBS). The cells were incubated for 7 d with a differentiation induction medium containing 10 µM test compounds and the medium was changed every 2 d. For further analysis, dorsomorphin (5 μM or 10 μM), L-748.337 (10 μM), and GW9662 (5 μM) were used.

### 2.2. Quantitative Real-Time RT-PCR

Total RNA was isolated using RiboEX (GeneAll Biotechnology, Seoul, Korea) and 1 μg of RNA was reverse-transcribed into cDNA using GoScript Reverse Transcription System (Promega Biotechnology, Madison, WI, USA). SYBR Green Master Mix (Roche Diagnostics GmbH, Mannheim, Germany) was used to quantitatively determine the transcript levels of genes on a StepOnePlus Real-Time PCR System (Thermo Fisher Scientific). The transcript levels of all the observed genes were normalized to those of glyceraldehyde 3-phosphate dehydrogenase (GAPDH), and ROX refernce dye (Thermo Fisher Scientific) was used as an experimental control. The nucleotide sequences of the primers used in this study are listed in Table S1.

### 2.3. Immunoblotting

The cell lysates were prepared using RIPA buffer (Thermo Fisher Scientific) via homogenization followed by centrifugation at 12,000 rpm for 10 min. The protein concentration was measured using a Pierce™ BCA Protein Assay Kit (Thermo Fisher Scientific). Subsequently, 5–10 µg of total protein was denatured by heating it at 95 °C for 5 min in 4× sample buffer (50 mM Tris, pH 6.8; 2% sodium dodecyl sulfate (SDS); 10% glycerol; 5% β-mercaptoethanol; 0.1% bromophenol blue), prior to being subjected to SDS–polyacrylamide gel electrophoresis (SDS-PAGE) on 8%, 10%, or 12% gels. After electrophoresis, the samples were transferred to a polyvinylidene difluoride membrane (Merck Millipore, Burlington, MA, USA) and blocked for 1 h with 5% bovine serum albumin (BSA) prepared in tris-buffered saline with tween 20 (TBS-T, 10 mM Tris-HCl, 150 mM NaCl, and 0.1% Tween 20). The membrane was consecutively rinsed thrice with TBS-T, followed by incubation for 18 h with 1:1000 dilutions of the following primary polyclonal antibodies: anti-vinculin (#13901), anti-PPARγ (#2443), anti-AMPK (#2532), anti-p-AMPK (#2531), anti-UCP1 (#14670), anti-PGC1α (#4259), anti-PKA (#4782), anti-ACC (#3676), anti-pACC (#3661) (Cell Signaling Technology, Danvers, MA, USA), and anti-FGF21 (MA5-32652) (Thermo Fisher Scientific) in 5% BSA prepared in TBS-T. After three washes, the membrane was incubated for 1 h with horseradish peroxidase-conjugated anti-mouse IgG or anti-rabbit IgG secondary antibodies (1:5000, Santa Cruz Biotechnology, Dallas, TX, USA) in 1% BSA prepared in TBS-T buffer. Chemiluminescence was detected using the Clarity™ ECL Western Blotting Substrate system (Bio-Rad Laboratories, Hercules, CA, USA) and imaged with LAS-4000 (GE Healthcare, Chicago, IL, USA). ImageJ was used for quantification.

### 2.4. Mitochondrial Staining

Differentiated 3T3-L1 were washed with PBS and incubated with complete medium containing 200 nM of MitoTracker™ Deep Red (Thermo Fisher Scientific) for 30 min. Images were obtained using an Operetta CLS High-Content Imaging System (Perkin Elmer, Waltham, MA, USA) and digitalized using harmony software (Perkin Elmer).

### 2.5. Animal Experiments

All experimental mice were housed in a specific pathogen-free facility at the Laboratory Animal Center, Daegu-Gyeongbuk Medical Innovation Foundation (DGMIF). All C57BL6/J mice were obtained from Orient Bio (Sungnam, Korea) and maintained and used in accordance with the Guidelines for the Care and Use of Laboratory Animals of the Laboratory Animal Center, DGMIF. The animal studies were conducted after obtaining approval from the institutional review board (IRB) of the DGMIF (approval number: DGMIF-18070202-00), and conformed to the ethics of animal experiments recommended by DGMIF. All efforts were made to minimize the suffering of the mice. Briefly, all mice were provided ad libitum access to a 60 kcal % fat diet (D12492, Research Diets, New Brunswick, NJ, USA) and water after being divided into the following experimental groups (*n* = 5 per group): saline; berberine (10 mg/kg/day); a low dosage of D-mannitol (D-mann LD; 250 mg/kg/day); and a high dosage of D-mannitol (D-mann HD; 500 mg/kg/day). Saline, berberine, and D-mannitol were administered daily for 3 weeks, following which the mice were anesthetized and euthanized via cervical dislocation. The inguinal white adipose tissue (iWAT) and interscapular brown adipose tissue (iBAT) were excised, rinsed with PBS, and stored at −80 °C for further analysis.

### 2.6. Statistical Analysis

All data—expressed as the mean ± SD—were compared using one-way ANOVA followed by Tukey’s post-hoc tests. The significance of the in vitro experiments was indicated as either * = *p* < 0.05 or ** = *p* < 0.01; for the in vivo experiment, significance was indicated as * = *p* < 0.05, ** = *p* < 0.005 and *** = *p* < 0.0001.

## 3. Results

### 3.1. D-Mannitol Induces Browning of 3T3-L1 Adipocytes

**Figure 1:**
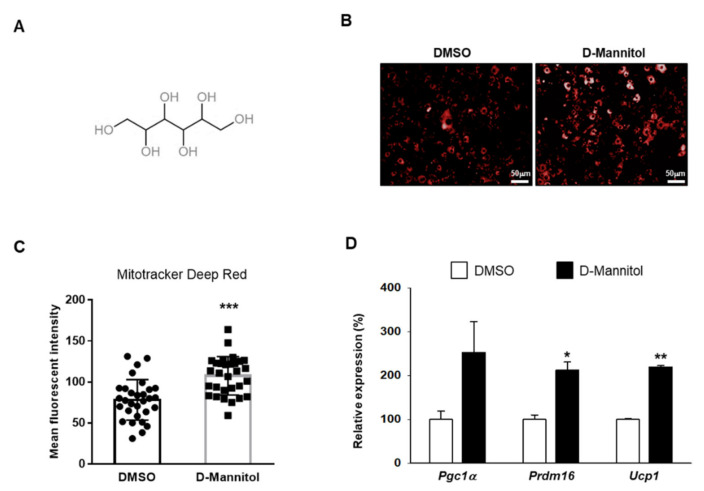
D-mannitol increased the mitochondrial content in differentiated adipocytes. The structure of D-mannitol (**A**). Adipocytes were treated with D-mannitol for 7 d and the mitochondrial contents were analyzed using MitoTracker™ Deep Red (**B**). The bar chart represents the mean fluorescent intensity of MitoTracker™ Deep Red-labeled mitochondria (*n* = 30) (**C**). All images were acquired using an Operetta CLS High-Content Imaging System and staining intensity was evaluated using Harmony software (**B**,**C**). The relative expression of brown fat-specific genes was analyzed following D-mannitol treatment (**D**). The significance of the expression of these genes in dimethyl sulfoxide (DMSO)- and D-mannitol-treated groups is shown; * = *p* < 0.05, ** = *p* < 0.01, and *** = *p* < 0.001.

As BAT contains a greater number of mitochondria than WAT, we attempted to identify a compound capable of increasing mitochondrial counts in the cell. Thus, 133 compounds were screened for their ability to induce the differentiation of 3T3-L1 preadipocytes, and the mitochondria content was measured based on the intensity of the mitotracker. Interestingly, we found that D-mannitol (Figure 1A) increased the mitochondrial content in the differentiated 3T3-L1 cellular model (Figure 1B,C), indicating that this molecule plays a role in mitochondrial biogenesis.

**Figure 2:**
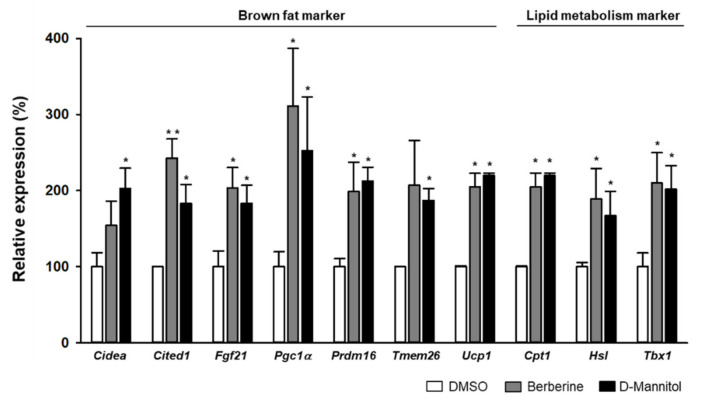
D-mannitol enhanced the expression of brown adipose tissue (BAT)-specific genes. The adipocytes were treated with 10 μM D-mannitol and the expression of brown cell-specific genes and lipid metabolism markers was measured via real-time RT-PCR. The cells treated with berberine (10 μM) were used as a positive control. The significance of target gene expression in DMSO- and compound-treated groups is shown; * = *p* < 0.05, and ** = *p* < 0.01.

To investigate the effect of D-mannitol on adipocyte differentiation, 3T3-L1 adipocytes were first treated with 10 μM D-mannitol and then the expression of brownfat-related genes was analyzed. Interestingly, D-mannitol dramatically increased the expression of PPARγ coactivator 1α (PGC1α)*,* PR domain containing 16 (PRDM16), and UCP1 (Figure 1D). When the transcript levels of the brown fat and lipid metabolism markers were measured, we found that the expression of genes associated with energy expenditure and thermogenesis was increased following the treatment of 3T3-L1 adipocytes with D-mannitol (Figure 2). The effect of D-mannitol was similar to that of berberine, a thermogenesis-activating compound [14]. In detail, the expression of UCP1 as well as that of other classical BAT marker genes, such as cell death-inducing DNA fragmentation factor alpha (Cidea), carnitine palmityl transferase 1 (CPT1), hormone-sensitive lipase (HSL), PGC1α, PRDM16, transmembrane protein 26 (Tmem26) and UCP1, and beige adipocyte markers, such as fibroblast growth factor 21 (FGF21), cbp/p300-interactiong transactivator 1 (Cited1) and T-box protein 1 (Tbx1), was dramatically increased. These results suggested that D-mannitol acts as a potential inducer of thermogenic programming by inducing the expression of BAT-related genes.

### 3.2. D-Mannitol Induces Brown Fat Phenotype via the AMPK Signaling Pathway

**Figure 3:**
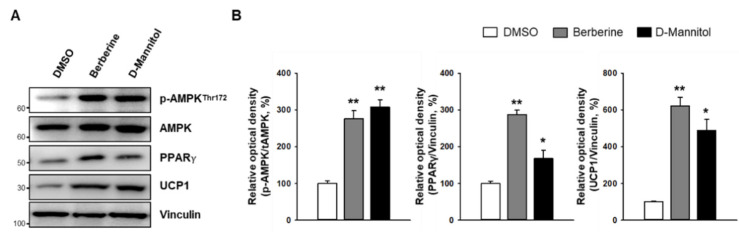
D-mannitol activates the AMPK pathway. The phosphorylation of AMPK and the expression of peroxisome proliferator-activated receptor gamma (PPARγ) and uncoupling protein 1 (UCP1) were measured via Western blotting after treatment with D-mannitol for 7 d (**A**). Band intensity was measured using ImageJ and ratios were calculated by normalizing against the indicated control (**B**). The significance of expression in DMSO- and compound-treated groups is shown; * = *p* < 0.05, and ** = *p* < 0.01.

In addition to increasing the expression of PGC1α—a critical transcriptional coactivator of UCP1 [15]—D-mannitol also increased the expression of UCP1 and peroxisome proliferator-activated receptor gamma (PPARγ) proteins (Figure 3). By regulating the expression of several genes, PPARγ enhanced the action of insulin and decreased blood glucose levels [15]. Furthermore, these results were more pronounced following D-mannitol treatment, which increased FGF21 expression (Figure 5C), further confirming the role of D-mannitol in thermogenic programming.

**Figure 4:**
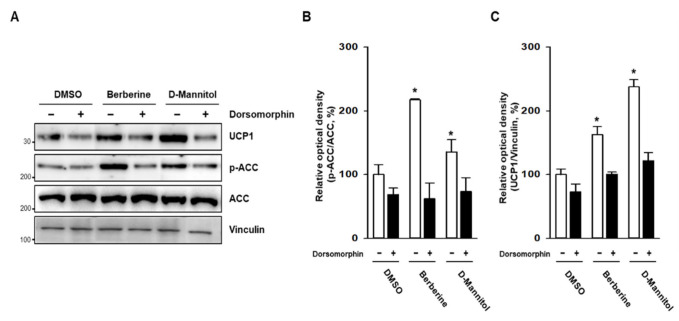
D-mannitol increased the expression of UCP1 in an AMPK-dependent manner. The adipocytes were treated with dorsomorphin (AMPK inhibitor; 10 μM) in the presence or absence of D-mannitol for 7 d, and the expression of UCP1 and acetyl-CoA carboxylase 1 (ACC) phosphorylation was measured via Western blotting (**A**), and the relative optical density was calculated using ImageJ (**B**,**C**). The significance of relative optical density in DMSO- and compound-treated groups is shown; * = *p* < 0.05.

In response to low-energy stress, AMPK increases catabolic activities to generate adenoine triphosphate (ATP) and inhibits anabolic activities, which require ATP. Upon phosphorylation at the threonine 172 residue, AMPK plays a critical role in regulating β-oxidation by phosphorylating and inhibiting the enzymes acetyl-CoA carboxylase 1 (ACC) and hydroxy-methyl-glutaryl-CoA reductase (HMGCR) [16]. To identify the possible mechanism underlying the browning effect of D-mannitol, we determined the phosphorylation levels of AMPK. Compared with berberine—a well-known AMPK activator [14]—D-mannitol dramatically increased the phosphorylation of AMPK at a similar level, and significantly induced the expression of PPARγ (Figure 3). Furthermore, D-mannitol dramatically increased the phosphorylation of ACC and the expression of UCP1 at a level similar to that observed when using berberine (Figure 4). Further, inhibition of AMPK by treatment with dorsomorphin (5 μM) abolished not only the expression of UCP1, but also the phosphorylation of ACC. These expression patterns indicated that D-mannitol induces the transformation of white adipocytes into beige adipocytes in addition to inducing thermogenesis in 3T3-L1 adipocytes via AMPK-mediated mechanisms.

### 3.3. D-Mannitol Induces Browning of White Adipocytes by Activating the β3-Adrenergic Receptor as Well as PGC1α and PKA

**Figure 5:**
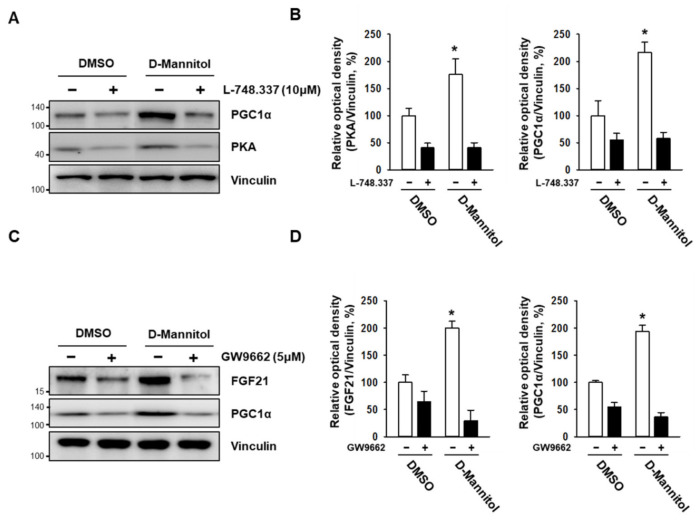
D-mannitol induced the generation of a brown fat-like phenotype in adipocytes via a β3-adrenergic receptor (β3-AR)- and PPARγ-dependent mechanism. The adipocytes were treated with L-748.337 (β3-AR antagonist; 10 μM) (**A**) and GW9662 (PPARγ antagonist) (5 μM) (**C**), and protein expression levels were determined via Western blotting. The relative optical density was calculated using ImageJ (**B**,**D**). The significance of protein expression in DMSO- and D-mannitol-treated groups is shown; * = *p* < 0.05.

Upon stimulation, several proteins including liver kinase B1 (LKB1), calcium/calmodulin-dependent protein kinase kinase (CaMKK), and the β3-adrenergic receptor induce the activation of AMPK [17]. The β3-adrenergic receptor, in particular, activates the AMPK signaling pathway [18] via PKA to promote the lipolysis of triglycerides stored in WAT and BAT. To investigate whether AMPK-dependent browning by D-mannitol is mediated by the β-adrenergic receptor and related signaling pathways, we treated 3T3-L1 preadipocytes with a β3-adrenergic receptor antagonist (L-748.337; 10 μM) in the presence or absence of D-mannitol (10 μM) during 7 d of differentiation, and monitored the expression of browning-related signaling proteins. D-mannitol increased the expression of PKA and PGC1α, whereas the expression of these proteins was dramatically decreased in the background of L-748.337 treatment (Figure 5A). These results indicated that the β3-adrenergic receptor–PKA axis plays a role in the generation of the D-mannitol-dependent browning phenotype of WAT.

It has been reported that the β3-adrenergic receptor activates PPARγ via the activation of extracellular signal-regulated kinase (ERK) [19]. Based on previous results that showed that D-mannitol increases the expression of PPARγ, we evaluated the role of PPARγ in D-mannitol-mediated browning. When we treated adipocytes with a PPARγ antagonist—GW9662 (5 μM)—in the presence of D-mannitol (10 μM) for 7 d of differentiation, the D-mannitol-induced increase in the expression of the brown fat markers FGF21 and PGC1α was dramatically abolished (Figure 5C). This suggested that PPARγ plays an important role in D-mannitol-induced browning.

### 3.4. D-Mannitol Induces the Browning of White Adipocytes in a Mouse Model

**Figure 6:**
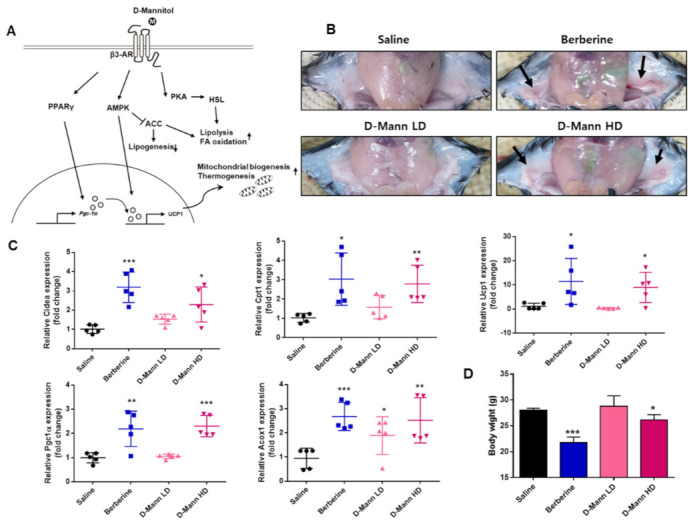
D-mannitol induced the expression of energy expenditure-related genes in inguinal white adipose tissue (iWAT). A schematic depicting the mechanism of action of D-mannitol (**A**). An image of mouse iWAT after 3 weeks of berberine and D-mannitol treatment. The arrows denote iWAT depots (**B**). High-fat diet-fed mice were treated as previously described. Gene expression profiles of energy expenditure and β-oxidation markers in iWAT were measured using real-time RT-PCR (**C**). The significant differences between saline- and compound-treated groups are shown; * = *p* < 0.05; ** = *p* < 0.005; *** = *p* < 0.0001. The body weights of the mice treated with berberine and with D-mannitol for 3 weeks were evaluated (**D**).

Next, we evaluated the effects of D-mannitol on browning in mice fed a high-fat diet. The mice were treated with berberine (10 mg/kg) or D-mannitol (D-mann LD; 250 mg/kg or D-mann HD; 500 mg/kg) for 3 weeks. The mice treated with berberine and D-mann HD experienced a significant decrease in mean body weight compared with saline-treated mice (Figure 6D). Additionally, imaging revealed that D-mannitol increased the transformation of white adipose tissue into brown tissue (Figure 6B). In the images of iWAT, the arrows that point towards the iWAT deposits in mice treated with berberine and D-mannitol HD reveal that the color of the iWAT was changed to brown (compared with the saline-treated group). However, mice treated with D-mannitol LD did not exhibit any significant changes in body weight or iWAT browning phenotype (Figure 6A,B). Consistent with these results, the expression of genes regulating mitochondrial thermogenesis and lipid oxidative function, such as Cidea, CPT1, UCP1, and PGC1α, was considerably increased in the iWAT of the berberine- and high dosage D-mannitol-treated groups (Figure 6C). Interestingly, no significant difference was observed between the mRNA levels of acyl-coenzyme A oxidase (ACOX1) in the control, and D-mannitol-treated 3T3-L1 adipocytes (data not shown). However, the transcript levels of ACOX1 in the berberine- and D-mannitol-treated groups were significantly increased, compared with those in the saline-treated group (Figure 6C). These results revealed that D-mannitol may increase energy consumption via lipid β-oxidation, thereby contributing to the browning of iWAT in vivo.

## 4. Discussion

In this study, we clearly demonstrated that D-mannitol may potentially induce brown fat-like phenotypes by significantly elevating brown fat-specific transcripts and proteins. The strategies that manipulate WAT into acquiring BAT-like characteristics and browning show potential as techniques that can be used to prevent and manage obesity. The D-mannitol treatment increased the expression of genes regulating brown fat-specific marker genes, such as Tbx1, Tmem26, and Cited1, as well as key transcription factors, such as PRDM16, and PGC1α, indicating D-mannitol-induced browning in the 3T3-L1 adipocytes. Notably, a considerable level of brite-specific gene expression was detected in the control 3T3-L1 adipocytes. This basal browning effect was mediated by dexamethasone and IBMX, which are involved in cell differentiation.

Our data strongly indicated that UCP1, a key protein found in beige adipocytes, which uncouples mitochondrial respiration for β-oxidation, was significantly upregulated by D-mannitol. Such increased UCP1 expression was accompanied by an increased mitochondrial content. Through the utilization of fatty acids, UCP1 in BAT not only regulates lipogenesis and lipolysis but also plays a role in heat generation [20]. Enhanced UCP1 expression was accompanied by elevated expression levels in PRDM16, which binds directly to PPARγ in order to facilitate fatty acid oxidation and thermogenesis, and acts as a master regulator in BAT development [21]. Moreover, D-mannitol-treated adipocytes showed enhanced levels of PPARγ, FGF21 and PGC1α, where elevated PGC1α and FGF21 expression levels were diminished following PPARγ inhibition. These results were further confirmed by a previous finding that reported that PPARγ increases energy expenditure, fat oxidation and excretion by increasing FGF21 [15]. Numerous reports have indicated that PPARγ activation induces white adipocyte cultures to acquire brown adipocyte-like characteristics [22,23,24]. Recently, Ohno et al. reported that agonistic PPARγ ligands induced a brown fat-specific gene profile in white adipocytes via the stabilization of the PRDM16 protein [25]. Collectively, PPARγ plays a dual role in adipocyte metabolism as follows: firstly, promoting the storage of excess fats in white adipocytes, and secondly its dissipation in brown adipocytes [26]. Together with the results of earlier studies, the finding of an increase in the expression of PPARγ following D-mannitol treatment suggests a possible role for UCP1 and PPARγ in D-mannitol-induced browning.

The investigation of underlying molecular mechanisms indicated that AMPK is associated with the inductive effects of D-mannitol during fat browning [27]. Our data show that D-mannitol significantly increased the expression and phosphorylation of AMPK, which was totally abolished by the inhibition of AMPK. Teperino et al. reported that AMPK activation using a hedgehog or a thyroid hormone increased glucose uptake in an insulin-independent manner, suggesting that D-mannitol played a role in regulating fuel utilization via AMPK-dependent sensing of energy levels. Moreover, the decrease in brown fat marker proteins, such as UCP1, subsequent to AMPK inhibition, reinforced our conclusion that D-mannitol induces browning via an AMPK-mediated pathway [28,29,30].

In addition, D-mannitol increased the expression of important mitochondrial markers, such as CPT1 and UCP1. It is well recognized that CPT1 and UCP1 increase mitochondrial activity, which enhances fatty acid oxidation in brown adipocytes [31]. CPT1 is involved in the rate-limiting step of the carnitine shuttle and in the transport of fatty acids to mitochondria, which is required for UCP1 activation. Therefore, the enhanced expression of these two proteins likely reflects the stimulation of fat oxidation by D-mannitol. D-mannitol also appears to stimulate lipolysis and suppress lipogenesis by increasing the expression levels of HSL and pACC. HSL is a key enzyme involved in lipolysis [32], while phosphorylation of ACC reduces the enzymatic activity of ACC, which plays a role in the rate-limiting step of fatty acid synthesis [33]. Therefore, the enriched expression of these proteins in white adipocytes indicates that D-mannitol plays a role in lipolysis and β-oxidation.

D-mannitol plays multiple regulatory roles, such as inducing the brown fat-like phenotype and enhancing lipid catabolism. First, D-mannitol acts as a β3-adrenergic receptor (β3-AR) agonist and consequently activates protein kinase A (PKA), which results in increasing lipolysis via the activation of HSL. It is well known that the β3-adrenergic receptor and its agonists regulate energy expenditure and promote the lipolysis of stored triglycerides in both white and beige adipocytes, via diverse signaling pathways [34].

β3-adrenergic receptor agonists, in particular, exert a wide range of effects, leading to increased energy expenditure, oxygen consumption and stimulation of insulin secretion, among others [35]. A significant reduction in the number of multiocular UCP1-positive brown adipocytes in β3-adrenergic receptor-null mice demonstrated that UCP1 expression in brown adipocytes of mice was regulated by the β3-adrenergic receptor [19,36]. The β3-adrenergic receptor regulates several key molecules involved in brown fat development, and thus its stimulation promotes the browning of white adipocytes [37]. Our results showed that increases in the expression of PGC1α and PKA were dramatically decreased after the β3-adrenergic receptor was inhibited, demonstrating that the effect of D-mannitol was mediated by β3-adrenergic receptor-dependent signaling. Although well-defined experimental results substantiated the role of D-mannitol in browning and lipolysis, the underlying mechanism needs to be studied in greater detail. In addition, additional investigations into the role of D-mannitol in glucose utilization and energy sensing may be warranted.

Here, we show that D-mannitol plays a critical role in the peroxisomal and mitochondrial oxidation of fatty acids in iWAT. In high-fat diet-fed mice, D-mannitol effectively upregulated the expression levels of Cidea, CPT1, UCP1, PGC1α, and ACOX1, which led to decreased body weight (Figure 6C,D). It is well authenticated that the β3-adrenergic receptor–PKA pathway, which upregulates UCP1 in mouse models, is the major mediator of thermogenesis in BAT [37]. In mouse BAT, PPARγ, PGC1α and PRDM16 are required for β3-adrenergic signaling-mediated induction of brown adipocytes [15,38]. Also, recent studies have shown that Cidea, which is not expressed in white adipocytes, is highly expressed in brown adipocytes in mice [39,40]. In addition, the expression of ACOX1, a rate-limiting enzyme of fatty acid β-oxidation in peroxisomes, was increased in the BAT of cold-induced mice [41], suggesting that D-mannitol affects the β3-adrenergic receptor-dependent browning mechanism in mouse models. However, despite the activation of energy consumption-related genes in iWAT, a significant increase in brown fat and lipid metabolism markers was not observed in iBAT without PGC1α (data not shown). Thus, further studies regarding the mechanisms underlying the regulation of browning in white adipose tissue by D-mannitol are felt to be required.

Considered together, the current study demonstrated that D-mannitol plays a role in not only fat browning but also maintaining energy homeostasis in cultured white adipocytes. Thus, D-mannitol shows potential as a new supplement that may be used to prevent obesity, with a focus on increasing energy expenditure by browning.

## Data Availability

Not applicable.

## Supplementary Materials

The following are available online at https://www.mdpi.com/article/10.3390/cells10040768/s1, Table S1: Sequences of primers used in this study for quantitative real-time PCR.

## References

[1] Kahn S.E., Hull R.L., Utzschneider K.M. (2006). Mechanisms Linking Obesity to Insulin Resistance and type 2 Diabetes. Nature.

[2] Gesta S., Tseng Y.H., Kahn C.R. (2007). Developmental Origin of Fat: Tracking Obesity to Its Source. Cell.

[3] Cannon B., Nedergaard J. (2004). Brown Adipose Tissue: Function and Physiological Significance. Physiol. Rev..

[4] Harms M., Seale P. (2013). Brown and Beige Fat: Development, Function and Therapeutic Potential. Nat. Med..

[5] Jörgensen J.A., Wu J., Mottaghy F.M., Schrauwen P., Van Marken Lichtenbelt W.D. (2013). Cold Acclimation Recruits Human Brown Fat and Increases Nonshivering Thermogenesis. J. Clin. Investig..

[6] Lecoultre V., Ravussin E. (2011). Brown Adipose Tissue and Aging. Curr. Opin. Clin. Natr. Metab. Care.

[7] Virtanen K.A., Lidell M.E., Orava J., Heglind M., Westergren R., Niemi T., Taittonen M., Laine J., Savisto N.J., Enerbäck S. (2009). Functional Brown Adipose Tissue in Healthy Adults. N. Engl. J. Med..

[8] Bonet M.L., Oliver P., Palou A. (2013). Pharmacological and Nutritional Agents Promoting Browning of White Adipose Tissue. Biochim. Biophys. Acta.

[9] Calvani R., Leeuwenburgh C., Marzetti E. (2014). Brown Adipose Tissue and the cold war against Obesity. Diabetes.

[10] Sellayah D., Bharaj P., Sikder D. (2011). Orexin Is Required for Brown Adipose Tissue Development, Differentiation, and Function. Cell Metab..

[11] Duluc L., Soleti R., Clere N., Andriantsitohaina R., Simard G. (2012). Mitochondria as Potential Targets of Flavonoids: Focus on Adipocytes and Endothelial Cells. Curr. Med. Chem..

[12] Izuta H., Shimazawa M., Tazawa S., Araki Y., Mishima S., Hara H. (2008). Effects of Chinese Propolis and Its Component, Chrysin, against Neuronal Cell Death via Inhibition of Mitochondrial Apoptosis Pathway in SH-SY5Y Cells. J. Agric. Food Chem..

[13] Anandhi R., Annadurai T., Anitha T.S., Muralidharan A.R., Najmunnisha K., Nachiappan V., Thomas P.A., Geraldine P. (2013). Antihypercholesterolemic and Antioxidative Effects of an Extract of the Oyster Mushroom, Pleurotus ostreatus, and Its Major Constituent, Chrysin, in Triton WR-1339-induced Hypercholesterolemic Rats. J. Physiol. Biochem..

[14] Zhang Z., Zhang H., Li B., Meng X., Wang J., Zhang Y., Yao S., Ma Q., Jin L., Yang J. (2014). Berberine Activates Thermogenesis in White and Brown Adipose Tissue. Nat. Commun..

[15] Puigserver P., Wu Z., Park C.W., Graves R., Wright M., Spiegelman B.M. (1998). A Cold-Inducible coactivator of Nuclear Receptors Linked to Adaptive Thermogenesis. Cell.

[16] Hardie D.G., Pan D.A. (2002). Regulation of Fatty Acid Synthesis and Oxidation by the AMP-Activated Protein Kinase. Biochem. Soc. Trans..

[17] Bijland S., Mancini S.J., Salt I.P. (2013). Role of AMP-Activated Protein Kinase in Adipose Tissue Metabolism and Inflammation. Clin. Sci..

[18] Yamada E., Lee T.W., Pessin J.E., Bastie C.C. (2010). Targeted Therapies of the LKB1/AMPK Pathway for the Treatment of Insulin Resistance. Future Med. Chem..

[19] Collins S. (2011). Beta-Adrenoceptor Signaling Networks in Adipocytes for Recruiting Stored Fat and Energy Expenditure. Front. Endocrinol..

[20] Fedorenko A., Lishko P.V., Kirichok Y. (2012). Mechanism of Fatty-Acid-Dependent UCP1 Uncoupling in Brown Fat Mitochondria. Cell.

[21] Villanueva C.J., Vergnes L., Wang J., Drew B.G., Hong C., Tu Y., Hu Y., Peng X., Xu F., Saez E. (2013). Adipose Subtype-Selective Recruitment of TLE3 or Prdm16 by PPARgamma Specifies Lipid Storage versus Thermogenic Gene Programs. Cell Metab..

[22] Sell H., Berger J.P., Samson P., Castriota G., Lalonde J., Deshaies Y., Richard D. (2004). Peroxisome Proliferator-Activated Receptor Gamma Agonism Increases the Capacity for Sympathetically Mediated Thermogenesis in Lean and ob/ob Mice. Endocrinology.

[23] Tai T.A., Jennermann C., Brown K.K., Oliver B.B., MacGinnitie M.A., Wilkison W.O., Brown H.R., Lehmann J.M., Kliewer S.A., Morris D.C. (1996). Activation of the Nuclear Receptor Peroxisome Proliferator-Activated Receptor Gamma Promotes Brown Adipocyte Differentiation. J. Biol. Chem..

[24] Vernochet C., Peres S.B., Davis K.E., McDonald M.E., Qiang L., Wang H., Scherer P.E., Farmer S.R. (2009). C/EBPalpha and the corepressors CtBP1 and CtBP2 Regulate Repression of Select Visceral White Adipose Genes during Induction of the Brown Phenotype in White Adipocytes by Peroxisome Proliferator-Activated Receptor Gamma Agonists. Mol. Cell. Biol..

[25] Ohno H., Shinoda K., Spiegelman B.M., Kajimura S. (2012). PPARgamma Agonists Induce a White-to-Brown Fat Conversion through Stabilization of PRDM16 Protein. Cell Metab..

[26] Roberts L.D., Murray A.J., Menassa D., Ashmore T., Nicholls A.W., Griffin J.L. (2011). The Contrasting Roles of PPARdelta and PPARgamma in Regulating the Metabolic Switch between Oxidation and Storage of Fats in White Adipose Tissue. Genome Biol..

[27] Pulinilkunnil T., He H., Kong D., Asakura K., Peroni O.D., Lee A., Kahn B.B. (2011). Adrenergic Regulation of AMP-Activated Protein Kinase in Brown Adipose Tissue In Vivo. J. Biol. Chem..

[28] Christoffolete M.A., Linardi C.C., de Jesus L., Ebina K.N., Carvalho S.D., Ribeiro M.O., Rabelo R., Curcio C., Martins L., Kimura E.T. (2004). Mice with Targeted Disruption of the DiO2 Gene Have Cold-Induced Overexpression of the Uncoupling Protein 1 Gene but Fail to Increase Brown Adipose Tissue Lipogenesis and Adaptive Thermogenesis. Diabetes.

[29] De Jesus L.A., Carvalho S.D., Ribeiro M.O., Schneider M., Kim S.W., Harney J.W., Larsen P.R., Bianco A.C. (2001). The type 2 Iodothyronine Deiodinase Is Essential for Adaptive Thermogenesis in Brown Adipose Tissue. J. Clin. Investig..

[30] Teperino R., Amann S., Bayer M., McGee S.L., Loipetzberger A., Connor T., Jaeger C., Kammerer B., Winter L., Wiche G. (2012). Hedgehog Partial Agonism Drives Warburg-Like Metabolism in Muscle and Brown Fat. Cell.

[31] Ali B.H., Adham S.A., Za’ A.M., Waly M.I., Yasin J., Nemmar A., Schupp N. (2015). Ameliorative effect of chrysin on adenine-induced chronic kidney disease in rats. PLoS ONE.

[32] Han L.K., Sumiyoshi M., Zhang J., Liu M.X., Zhang X.F., Zheng Y.N., Okuda H., Kimura Y. (2003). Anti-Obesity Action of Salix Matsudana Leaves (Part 1). Anti-Obesity Action by Polyphenols of Salix Matsudana in High Fat-Diet Treated Rodent Animals. Phytother. Res..

[33] Liu J.F., Ma Y., Wang Y., Du Z.Y., Shen J.K., Peng H.L. (2011). Reduction of Lipid Accumulation in HepG2 Cells by Luteolin Is Associated with Activation of AMPK and Mitigation of Oxidative Stress. Phytother. Res..

[34] Cao W., Medvedev A.V., Daniel K.W., Collins S. (2001). Beta-Adrenergic Activation of p38 MAP Kinase in Adipocytes: cAMP Induction of the Uncoupling Protein 1 (UCP1) Gene Requires p38 MAP Kinase. J. Biol. Chem..

[35] Seale P., Conroe H.M., Estall J., Kajimura S., Frontini A., Ishibashi J., Cohen P., Cinti S., Spiegelman B.M. (2011). Prdm16 Determines the Thermogenic Program of Subcutaneous White Adipose Tissue in Mice. J. Clin. Investig..

[36] Bachman E.S., Dhillon H., Zhang C.Y., Cinti S., Bianco A.C., Kobilka B.K., Lowell B.B. (2002). betaAR Signaling Required for Diet-Induced Thermogenesis and Obesity Resistance. Science.

[37] Cao W., Daniel K.W., Robidoux J., Puigserver P., Medvedev A.V., Bai X., Floering L.M., Spiegelman B.M., Collins S. (2004). p38 Mitogen-Activated Protein Kinase Is the Central Regulator of Cyclic AMP-Dependent Transcription of the Brown Fat Uncoupling Protein 1 Gene. Mol. Cell. Biol..

[38] Lasar D., Rosenwald M., Kiehlmann E., Balaz M., Tall B., Opitz L., Lidell M.E., Zamboni N., Krznar P., Sun W. (2018). Peroxisome Proliferator Activated Receptor Gamma Controls Mature Brown Adipocyte Inducibility through Glycerol Kinase. Cell Rep..

[39] Petrovic N., Walden T.B., Shabalina I.G., Timmons J.A., Cannon B., Nedergaard J. (2010). Peroxisome Proliferator-Activated Receptor Gamma (PPARgamma) Activation of Epididymally Derived White Adipocyte Cultures Reveals a Population of Thermogenically Competent, UCP1-Containing Adipocytes Molecularly Distinct from Classic Brown Adipocytes. J. Biol. Chem..

[40] Zhou Z., Yon Toh S., Chen Z., Guo K., Ng C.P., Ponniah S., Lin S.C., Hong W., Li P. (2003). Cidea-Deficient Mice Have Lean Phenotype and Are Resistant to Obesity. Nat. Genet..

[41] Zhang Y., Guo H., Deis J.A., Mashek M.G., Zhao M., Ariyakumar D., Armien A.G., Bernlohr D.A., Mashek D.G., Chen X. (2014). Lipocalin 2 Regulates Brown Fat Activation via a Nonadrenergic Activation Mechanism. J. Biol. Chem..

