# Supplementary material for: D-Mannitol Induces a Brown Fat-like Phenotype via a β3-Adrenergic Receptor-Dependent Mechanism
Source: Cells. 2021 Mar 31;10(4):768. doi: 10.3390/cells10040768 (PMC8066535; doi:10.3390/cells10040768)
Supplement: Supplementary file 1 [file cells-10-00768-s001.pdf]

Table 1. Sequences of primers used in this study for quantitative real-time PCR.

Gene	Forward	Reverse
Cidea	CGGGAATAGCCAGAGTCACC	TGTGCATCGGATGTCGTAGG
Cited1	GCGGTAAAAGATCGCAAGGC	TTGTAGAAGGGGTGGCAGTA
Cpt1	CTGAGCCATGAAGCCCTCAA	CACACCCACCACCACGATAA
Fgf21	CGTCTGCCTCAGAAGGACTC	TCTACCATGCTCAGGGGGTC
Hsl	CGGTG CGAAAAGGCAAGAT	GCAAAGACATTAGACAGCCGC
Pgc-1α	ATGTGCAGCCAAGACTCTGTA	CGCTACACCACTTCAATCCAC
Prdm16	CCCCACATTCCGCTGTGAT	CTCGCAATCCTTGCACTCA
Tbx1	CGAATGTTCCCCACGTTCCA	GTCTACTCGGCCAGGTGTAG
Tmem26	GAAACCAGTATTGCAGCACCC	CCAGACCGGTTCACATACCA
Ucp1	CCTGCCTCTCTCGGAAACAA	GTAGCGGGGTTTGATCCCAT
